# Expression of Concern: Assessment of time management practice and associated factors among primary hospitals employees in north Gondar, northwest Ethiopia

**DOI:** 10.1371/journal.pone.0326894

**Published:** 2025-06-23

**Authors:** The PLOS One Editors

The *PLOS One* Editors issue an Expression of Concern to alert readers that this article [1] was identified as one of a series of submissions for which we have concerns about authorship and adherence to the journal’s first and second publication criteria. In addition, the article [1] appears to report the same study and results as an article published later by the same author group [2].

Contrary to the declaration in the Data Availability statement, the original raw data files supporting the article’s results were not provided with the article, and the authors have not provided these data following editorial request. As the original raw data files supporting the article’s results have not been made available, this article [1] does not comply with the PLOS Data Availability policy.

We regret that these issues were not addressed prior to the article’s publication.
