# Supplementary material for: Assessment of time management practice and associated factors among primary hospitals employees in north Gondar, northwest Ethiopia
Source: PLoS One. 2020 Jan 17;15(1):e0227989. doi: 10.1371/journal.pone.0227989 (PMC6968858; doi:10.1371/journal.pone.0227989)
Supplement: S1 File — (DOCX) [file pone.0227989.s001.docx]

## “S1 file” : Questionnaires sheet

**General Instructions**

- No need of writing your name
- In all cases where answer options are available please tick (√) in the box provided
- For blank spaces provided write the answer over there.
- For scale typed questions please circle your preferred level of agreement

Thank you in advance for your honest cooperation.

**Part I: Demographic Information**

| **S.no** | **Question** | **Coding Categories** | | | |
| --- | --- | --- | --- | --- | --- |
| 1 | Sex | 1. Male | | 2. Female | |
| 2 | Age (years) |  | | | |
| 3 | Marital status | 1. Single | 2. Married | 3. Divorced | 4.widowed |
| 4 | Education level (specify) | 1.Diploma and below | 2. Degree | 3. MSC and above | |
| 5 | Work experience in years (specify) |  | | | |
| 6 | Residence (specify) | 1. High land 2. Low land | | | |
| 8 | Profession (specify) |  | | | |

**Part II: Time management factors**

**Instruction:** There are statements about factors affecting time management practices and each statement has five alternatives with five point scale. Read each item carefully and circle:

1= If you are **strongly disagreed** about the statement.

2= If you are **disagreed** about the statement.

3= If you are **neither agreed** nor **disagreed (neutral)** about the statement.

4= If you are **agreed** about the statement.

5= If you **strongly agreed** about the statement.

| **A** | **Personal Factor**s | Five point Likert scale | | | | |
| --- | --- | --- | --- | --- | --- | --- |
|  | **Variable 01: Procrastination** |  |  |  |  |  |
| 1 | I say "no" to others when I have a scheduled task | 1 | 2 | 3 | 4 | 5 |
| 2 | I don’t wait until the last minute to do things | 1 | 2 | 3 | 4 | 5 |
| 3 | I agree with the principle that the best time to do something is usually now | 1 | 2 | 3 | 4 | 5 |
| 4 | I set personal deadlines and stick to them | 1 | 2 | 3 | 4 | 5 |
|  | **Variable 02: Time waster** |  | | | | |
| 5 | I find it is easy to concentrate and get focused on a task | 1 | 2 | 3 | 4 | 5 |
| 6 | I take action to minimize interruptions or intrusions on my time | 1 | 2 | 3 | 4 | 5 |
| 7 | I avoid meetings that are not important and are time wasters | 1 | 2 | 3 | 4 | 5 |
| 8 | I avoid spending a lot of time on routine and trivial things. | 1 | 2 | 3 | 4 | 5 |
| 9 | I often avoid spend time socializing instead of working(internet, alcohol, TV) | 1 | 2 | 3 | 4 | 5 |
|  | **Variable 03: Punctuality** |  | | | | |
| 10 | I always enter to work on regular time | 1 | 2 | 3 | 4 | 5 |
| 11 | I always leave work on regular time | 1 | 2 | 3 | 4 | 5 |
| **B** | **Administrative and Organizational factors** |  | | | | |
|  | **Variable** **04: Organization policy and strategy** |  | | | | |
| 12 | There is ways employees are informed about the organization’ policies | 1 | 2 | 3 | 4 | 5 |
| 13 | There is ways the organization’s policies are put into practice | 1 | 2 | 3 | 4 | 5 |
| 14 | The way my boss takes care of the complaints of his/her employees | 1 | 2 | 3 | 4 | 5 |
|  | **Variable** 0**5**: **Performance appraisal** |  | | | | |
| 15 | My performance appraisal is conducted on time every 6month | 1 | 2 | 3 | 4 | 5 |
| 16 | I always receive a copy of my 6month’s performance appraisal | 1 | 2 | 3 | 4 | 5 |
| 17 | I feel that I am treated fairly compared with colleagues in my organization who have similar qualifications and who have similar work experience. | 1 | 2 | 3 | 4 | 5 |
|  | **Variable 06:**  **Work environment** |  | | | | |
| 18 | The hospital has adequate equipment’s to perform my job properly | 1 | 2 | 3 | 4 | 5 |
| 19 | The hospital has facilities which are clean and up-to-date. | 1 | 2 | 3 | 4 | 5 |
| 20 | In my Hospital office conditions are comfortable for work | 1 | 2 | 3 | 4 | 5 |
| 21 | As an individual I have adequate personal space in my department | 1 | 2 | 3 | 4 | 5 |
| 22 | The hospital provides adequate materials for work | 1 | 2 | 3 | 4 | 5 |
|  | **Variable** 0**7**: **Compensation & benefit** |  | | | | |
| 23 | My Salary is adequate for my living expenses | 1 | 2 | 3 | 4 | 5 |
| 24 | The performance recognition system in my organization is as good as in most other organizations | 1 | 2 | 3 | 4 | 5 |
| 25 | There are adequate financial rewards for those who work here | 1 | 2 | 3 | 4 | 5 |
|  | **Variable** 0**8**: **Recognition and Promotion** |  | | | | |
| 26 | The way I am noticed when I do a good job is appropriate | 1 | 2 | 3 | 4 | 5 |
| 27 | There is ways promotions are given out on this job | 1 | 2 | 3 | 4 | 5 |
| 28 | There is a chance to be recognized for the accomplishments on the job | 1 | 2 | 3 | 4 | 5 |
| 29 | There is a chance to recognize staff successes whatever small it would be | 1 | 2 | 3 | 4 | 5 |
| **C** | **Employees performance** |  | | | | |
|  | **Variable 09: Planning** |  | | | | |
| 30 | I plan my daily activities | 1 | 2 | 3 | 4 | 5 |
| 31 | I estimate the time it takes for my daily activities | 1 | 2 | 3 | 4 | 5 |
| 32 | I am able to meet deadlines without rushing at the last minute | 1 | 2 | 3 | 4 | 5 |
| 33 | I seek quality work in my job | 1 | 2 | 3 | 4 | 5 |
|  | **Variable 10: Implementation** |  | | | | |
| 34 | I start work immediately I enter office | 1 | 2 | 3 | 4 | 5 |
| 35 | I implement daily tasks on schedule | 1 | 2 | 3 | 4 | 5 |
| 36 | I assess my daily tasks accomplishment before I leave work | 1 | 2 | 3 | 4 | 5 |
|  | **Variable 11: Responsibility** |  | | | | |
| 37 | I am responsible for my work | 1 | 2 | 3 | 4 | 5 |
| 38 | I am fulfilling my responsibility at work | 1 | 2 | 3 | 4 | 5 |
| 39 | I am responsible for the works of others | 1 | 2 | 3 | 4 | 5 |

**Part III: Employees Time management practice measuring items**

There are statements about time management practices in an organization, and each

Statement has five alternatives with five point scale. Read each item carefully and circle:

1= If you **strongly disagree** about the statement.

2= If you **disagree** about the statement.

3= If you **neither agree nor disagree (neutral)** about statement.

4= If you **agree** about the statement

5= If you **strongly agree** about the statement.

| **No.** | **Time management practice measurining items** | Five point Likert scale | | | | |
| --- | --- | --- | --- | --- | --- | --- |
| 1 | I have short and long term goals | 1 | 2 | 3 | 4 | 5 |
| 2 | I have detained list of priority tasks | 1 | 2 | 3 | 4 | 5 |
| 3 | I prioritize tasks based on importance | 1 | 2 | 3 | 4 | 5 |
| 4 | I do a schedule for each activity | 1 | 2 | 3 | 4 | 5 |
| 5 | I execute tasks and give delegation as appropriate | 1 | 2 | 3 | 4 | 5 |
