# Supplementary material for: Assessment of time management practice and associated factors among primary hospitals employees in north Gondar, northwest Ethiopia
Source: PLoS One. 2020 Jan 17;15(1):e0227989. doi: 10.1371/journal.pone.0227989 (PMC6968858; doi:10.1371/journal.pone.0227989)
Supplement: Attachment — Submitted filename: Response to reviewer final.docx [file pone.0227989.s003.docx]

Rebuttal letter	Date: November/11/2019

PONE-D-19-24146

**Assessment of time management practices and associated factors among employees of primary hospitals in north Gondar, northwest Ethiopia**

**Erkihun Tadesse**

**PLOS ONE**

Dear all,

We would like to thanks for these constructive, building and improvable comments on this manuscript that would improve substance and content of the manuscript. We considered each comments and clarification questions of editors and reviewers on the manuscript thoroughly. Our point-by-point responses for each comment and questions are described in detailed on the following pages. Further, the details of changes were shown by track changes in the supplementary document attached.

| **Editor questions/comments** | **Response** |
| --- | --- |
| **Abstract**  The introduction in the abstract section need to be shorten and depict time management and purpose of the study in a clear fashion. In general, this section must show the entire study with limited words if possible with in one page. | Thanks editor for your constructive comment. We have corrected this section by depicting time management practice and purpose of the stud in the main document according to your query. |
| **Methodology**  I haven't seen a single statement that shows the operational definition of your study variable. It would be also very useful to include the dependent and independent variable and its operational definition to provide a clear picture of this study. | Dear editor thanks for your constructive comments. We have included the dependent and independent variable, and its operational definition in the main document as shown in the truck number from 146 – 192. |
| **Result**  The Result section appears to be improved at a different perspective, however, I recommend you to separate the subtopic organization policy, employment performance and personal factors. | Thank you editor for the comment. We have separated the organization related factors of respondents, Employees performance related factors and personal related factors of respondents separately in the main document. |
| **Discussion**  The discussion well written and improved significantly. However, still you need to keep the flow of the write up based on the analysis on the result section, which will help you maintain the coherence of statement in alignment with the result section. | Thank you editor for the comment. We have arranged the discussion section according to the result section in the main document. |
