# Supplementary material for: Assessment of time management practice and associated factors among primary hospitals employees in north Gondar, northwest Ethiopia
Source: PLoS One. 2020 Jan 17;15(1):e0227989. doi: 10.1371/journal.pone.0227989 (PMC6968858; doi:10.1371/journal.pone.0227989)
Supplement: Attachment — Submitted filename: Response to reviewer 3@.docx [file pone.0227989.s004.docx]

Rebuttal letter	Date: January/01/2020

PONE-D-19-24146

**Assessment of time management practice and associated factors among primary hospitals employees in north Gondar, northwest Ethiopia**

**Erkihun Tadesse**

**PLOS ONE**

Dear all,

We would like to thanks for these constructive, building and improvable comments on this manuscript that would improve substance and content of the manuscript. We considered each comments and clarification questions of editors and reviewers on the manuscript thoroughly. Our point-by-point responses for each comment and questions are described in detailed on the following pages. Further, the details of changes were shown by track changes in the supplementary document attached.

| **Editor questions/comments** | **Response** |
| --- | --- |
| **Introduction**  I advise you to remove the last statement after the purpose of the study that appears to be a recommendation and the quote to refer previous study, you don’t have a tangible assertion whether the same topic conducted or not in Ethiopia setting.  Please remove the following paragraph “which was not studied previously. Thus the findings from the study would provide valid information for policy makers, managers and stakeholders for taking appropriate action to improve time management practice.” | Thanks editor for your constructive comment. We have removed the paragraph “which was not studied previously. Thus the findings from the study would provide valid information for policy makers, managers and stakeholders for taking appropriate action to improve time management practice.” Expressed as **Therefore, this study aimed to assess time management practices and associated factors among employees of primary hospitals in North Gondar Zone.** |
| **Discussion**  Your arguments need to have reasonable evidence. Your assumption to elucidate the discrepancy originally inferred based on what you thought about it, which is not scientifically sound even if your arguments are correct. There is plenty of research out there that would support your assumption, hence I recommend you to rewrite this section using references that bolster your assertion. | Thank you editor for the comment. We have supported the arguments/assumptions using references and corrected according to your inquiry in the main documents. |
| **Conclusion**  Make sure your conclusion and recommendation based on merely on the finding of this study. Don’t try to additional points out this study context. | Thank you editor for constructive comments. We have presented the conclusion and recommendation based on the finding of this study and corrected according to your inquiry. |
